# Effect of mini-course training in communication and teamwork on non-technical skills score in emergency residents: a prospective experimental study

**DOI:** 10.1186/s12909-023-04507-7

**Published:** 2023-07-25

**Authors:** Pitsucha Sanguanwit, Thanet Kulrotwichit, Welawat Tienpratarn, Natsinee Athinartrattanapong, Thavinee Trainarongsakul, Chuenruthai Angkoontassaneeyarat

**Affiliations:** Department of Emergency Medicine, Faculty of Medicine, Ramathibodi Hospital, Mahidol University, 270 Rama VI Rd., Ratchathewi, Bangkok, 10400 Thailand

**Keywords:** Communication, Medical education, Non-technical skills, Teamwork, Emergency residents

## Abstract

**Background:**

Non-technical skill (NTS) teaching is a recent development in medical education that should be applied in medical education, especially in medical specialties that involve critically ill patients, resuscitation, and management, to promote patient safety and improve quality of care. Our study aimed to compare the effects of mini-course training in NTS versus usual practice among emergency residents.

**Methods:**

In this prospective (non-randomized) experimental study, emergency residents in the 2021–2022 academic year at Ramathibodi Hospital, a tertiary care university hospital, were included as participants. They were categorized into groups depending on whether they underwent a two-hour mini-course training on NTS (intervention group) or usual practice (control group). Each participant was assigned a mean NTS score obtained by averaging their scores on communication and teamwork skills given by two independent staff. The outcome was the NTS score before and after intervention at 2 weeks and 16 weeks.

**Results:**

A total of 41 emergency residents were enrolled, with 31 participants in the intervention group and 10 in the control group. The primary outcome, mean total NTS score after 2 weeks and 16 weeks, was shown to be significantly better in intervention groups than control groups (25.85 ± 2.06 vs. 22.30 ± 2.23; P < 0.01, 28.29 ± 2.20 vs. 23.85 ± 2.33; P < 0.01) although the mean total NTS score did not differ between the groups in pre-intervention period. In addition, each week the NTS score of each group increased 0.15 points (95% CI: 0.01–0.28, P = 0.03), although the intervention group showed greater increases than the control (0.24 points) after adjustment for time (95% CI: 0.08–0.39, P < 0.01).

**Conclusion:**

Emergency residents who took an NTS mini-course showed improved mean NTS scores in communication and teamwork skills versus controls 2 weeks and 16 weeks after the training. Attention should be paid to implementing NTS in the curricula for training emergency residents.

**Trial registration:**

This trial was retrospectively registered in the Thai Clinical Trial Registry on 29/11/2022. The TCTR identification number is TCTR20221129006.

## Background

Non-Technical Skills (NTS), also referred to as soft skills, are the set of cognitive and social skills that are not related to a specific job task but are necessary for optimal performance [1, 2]. NTS comprises seven core categories, including Situational Awareness, Decision-Making, Communication, Teamwork, Leadership, Managing Stress, and Coping with Fatigue. Training in NTS was first implemented in the aviation industry as part of Crew Resource Management (CRM)[1] to reduce human error and promote safety. Its success has led to the acceptance of NTS training in many industries that must maintain high standards, including medical care.

NTS training has become an area of particular interest in medical education and many medical specialties [3–5] because studies showed its ability to promote patient safety and quality of care in critical situations [6, 7]. Tools such as Anesthetists’ Non-technical Skill (AS-NTS) [8] and Non-Technical Skills for Surgeons (NOTSS) [3] were validated for developing professional competencies and introducing effective models for teaching and evaluating NTS.

Even though the tools used to measure NTS have specifications based on medical specialties, they are also applicable in other specialties and situations. For example, NOTSS was initially developed and validated for surgeons in the operating room [9], but a study reported using NOTSS to measure NTS in trauma resuscitation stimulations in a team of emergency medicine and surgical residents [3, 10]. There are seven core categories in which NTS should be applied in healthcare [11].

NTS plays an important role in improving patient care in the emergency department, particularly in communication [12] and teamwork [6]. Teamwork is a skill required for a team to be successful in resuscitating critically ill patients [13]. Communication skills are necessary not only for consultation with other specialties [14] but also for sharing and giving information to patients and relatives, increasing patient satisfaction and decreasing complaints [15].

We aimed to compare the effect of mini-course training for NTS versus usual practice in communication and teamwork scores before, after 2 weeks and after 16 weeks among emergency residents. Two main reasons for choosing only communication and teamwork. First, communication and teamwork are frequently used in routine practice in the emergency department. Second, It’s easily measurable by direct observation.

## Methods

### Study design

This study was a prospective non-randomized experimental study comparing NTS scores between emergency residents receiving mini-course training on non-technical skills (the intervention group) versus usual practice (the control group) in communication and teamwork scores measured before the course and 2 weeks and 16 weeks after. This study was approved by the Faculty of Medicine, Committee on Human Rights Related to Research Involving Human Subjects, at Mahidol University’s Ramathibodi Hospital on April 29, 2021 (IRB COA. MURA2021/360).

### Study population

This study was conducted at the Emergency Medicine Department of Ramathibodi Hospital, a university-affiliated tertiary care hospital in Bangkok, Thailand. We assessed the eligibility of all emergency residents in the academic year 2021–2022. We excluded emergency residents who could not undergo the follow-up NTS score evaluation in communication and teamwork at 2 weeks and 16 weeks after the training. All participants provided signed informed consent.

### Study intervention

Before starting the mini-course training NTS, we collected baseline characteristics including age, post-graduate year, academic year (resident), marital status, and history of prior communication or teamwork training. All participants underwent an NTS score evaluation 2 months before receiving the intervention. Those who did not participate in the formal training underwent usual practice, which at this institution consists of learning on the job and may receive feedback from staff during daily practice by chance. However, we had the curriculum of formal regular feedback 2 times per year about NTS from academic staff.

In our study, we included two emergency staff members, not involved in the study analysis, who were trained in NTS score assessment by the investigators. We sent an evaluation form to the assessors that included all participants. We did not label for intervention or control group to assessors. They each observed the participant’s service in the emergency room for around 15–30 min and independently assigned the NTS score; then, we used the mean score of the two assessors for the overall NTS score of each participant. We used simulation scenarios to examine the agreement of the two assessors in NTS score assessment for the emergency residents. The level of inter-rater agreement in this reliability test was fair (Cohen’s kappa statistic 0.4, agreement 68.75%).

Emergency residents underwent either mini-course training in NTS communication and teamwork group or usual practice in September 2021 based on their convenience. The mini-course training group had one hour of lectures on communication and teamwork skills, then another hour of simulation and debriefing for practice on communication and teamwork skills. The lecture and simulation on communication and teamwork were delivered by five academic staff members certified in NTS teaching. We added education methods, including self-reflection, feedback, and debriefing from team learning to self-practice in NTS.

**Fig. 1:**
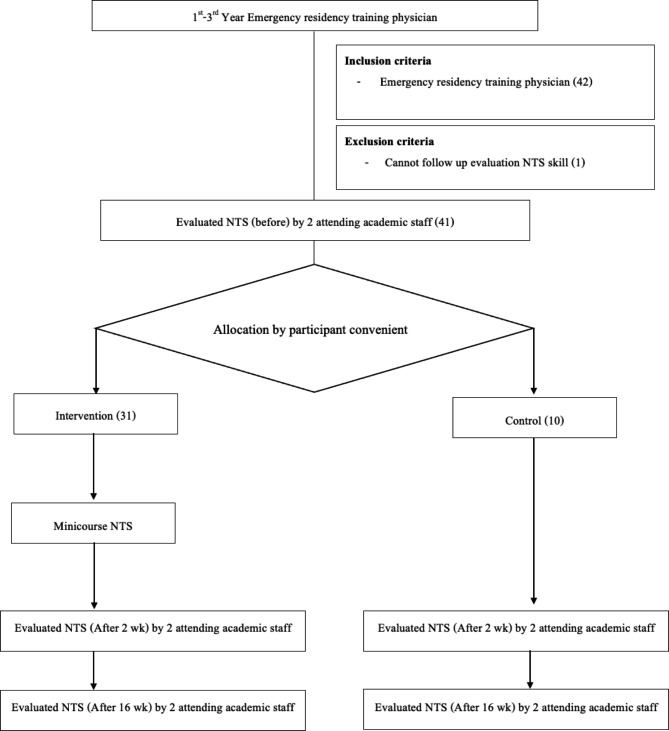
Study protocol

In the first simulation, the objective was interfacility communication for inter-hospital transfers of respiratory failure patients in whom SAR-CoV-2 was detected. The second simulation concerned managing communication and teamwork in emergency department care for traumatic hypotension. The protocol is illustrated in Fig. 1.

### Study outcome

The primary outcome was the mean total NTS score 2 weeks and 16 weeks after the NTS mini-course training. Secondary outcomes included the mean communication score, the mean teamwork score, and the scores of each component 2 weeks and 16 weeks after the NTS training. The mean total NTS score was calculated from the sum of the mean communication skill score and teamwork skill score. Communication and teamwork skills were assessed using integration scales adapted from the essential NTS. We designed to evaluate the immediate and delayed effects of the intervention which we adapted follow up time from Gorgas et al. [16].

The scoring system was adapted from Kodate et al. [11] in the communication and teamwork domains, each of which had four categories (Table 1). We used the following rating scale for evaluation: 1 = poor (performance endangered or potentially endangered patient safety, serious remediation is required); 2 = marginal (performance indicated cause for concern, considerable improvement is needed); 3 = acceptable (performance was of a satisfactory standard but could be improved); and 4 = good (performance was of a consistently high standard, enhancing patient safety; it could be used as a positive example for others). These scores were aggregated to provide an overall score that had range of 4–16 in each domain. Higher scores indicated better NTS.

**Table 1:** Core non-technical skills in communication and team work [10, 11]

Skill	Item
Communication skills	• Giving information clearly and concisely
	• Including context and intent
	• Receiving information
	• Identifying and tackling barriers to communication
Teamwork skills	• Supporting others
	• Solving conflicts
	• Exchanging information
	• Coordinating activities

### Sample size and statistical analysis

We estimated sample size based on Yule et al. [17] assuming a mean score of NOTSS before and after coaching of 10.8 and 13.8, respectively, with an SD of 4. The maximum score was 16. This study needed sixteen participants to detect potential differences between the two groups, with a two-sample paired-mean test α of 0.05 and a power of 0.8.

For comparison of baseline characteristics between the two groups, continuous variables were reported using mean ± SD for the normal distribution or medians and interquartile ranges in non-parametric tests. Tests for differences employed independent t-tests, or Mann–Whitney U tests as appropriate. For categorical variable data represented with frequency and proportion, tests for difference used Chi-squared test or Fisher’s exact test as appropriate.

The primary outcome and secondary outcome were analyzed using repeated data analysis to detect changes in NTS scores before and 2 weeks and 16 weeks after the training. All statistical analysis was conducted using STATA version 16.1 (StataCorp, College Station, TX, USA).

## Results

A total of 41 emergency resident physicians were enrolled; those who participated in the mini-course training made up the intervention group of 31 participants; the remaining 10 participants (the control group) underwent usual practice.

Table 2 shows the baseline characteristic data in each group. Overall, age, marital status, years of post-graduate experience, academic year, and rates of having prior communication and teamwork training were statistically similar between groups. Table 3 demonstrates that the primary outcome of our study, mean total NTS score after 2 weeks and 16 weeks, was significantly better in the mini-course training group than the control group (25.85 ± 2.06 vs. 22.30 ± 2.23; P < 0.01, 28.29 ± 2.20 vs. 23.85 ± 2.33; P < 0.01); the mean total NTS score did not differ between the groups in the pre-intervention period.

**Table 2:** Demographic and descriptive data by group

Characteristic	Total (N = 41)	Mini-course training (N = 31)	Usual training (N = 10)	P-value
**Age (years)**	28.80 ± 1.23	28.84 ± 1.30	28.70 ± 1.06	0.761
**Marital status** Single Married	38 (92.68%) 3 (7.32%)	28 (90.32%) 3 (9.68%)	10 (100%) 0 (0%)	0.42
**years of post-graduate experience**	2.63 ± 0.77	2.68 ± 0.65	2.5 ± 1.08	0.53
**Academic year (Resident)** R1 R2 R3	14 (34.15%) 13 (31.71%) 14 (34.15%)	11 (35.48%) 10 (32.26%) 10 (32.26%)	3 (30%) 3 (30%) 4 (40%)	1.00
Prior training* - yes - no	17 (41.46%) 24 (58.54%)	15 (48.39%) 16 (51.61%)	2 (20%) 8 (80%)	0.11

*Prior communication and teamwork training.

**Table 3:** Comparison of effects on training on mean NTS score (primary outcome)

Element	Total (N = 41)	Mini-course training (N = 31)	Usual training (N = 10)	P-value
**Primary outcome: Mean total non-technical skills score**				
Pre-intervention	20.49 ± 1.73	20.26 ± 1.45	21.20 ± 2.36	0.14
2-week	24.99 ± 2.59	25.85 ± 2.06	22.30 ± 2.23	< 0.01
16-week	27.21 ± 2.93	28.29 ± 2.20	23.85 ± 2.33	< 0.01

The secondary outcome of our study shows that the mean communication skills score prior to the intervention was significantly lower in the mini-course training group than the usual training group (9.76 ± 0.72 vs. 10.50 ± 1.08; P = 0.02); the mini-course training groups had significantly better score after 2 weeks and 16 weeks than the usual training groups (12.76 ± 1.12 vs. 11.05 ± 1.19; P < 0.01, 13.97 ± 1.24 vs. 11.75 ± 1.21; P < 0.01). Evaluation of the communication skills subdomain score revealed that giving information clearly and concisely, including context and intent, receiving information, identifying and tackling barriers to communication, all showed improved scores in the mini-course training group after 2 weeks and 16 weeks compared with the usual training group (Table 4).

**Table 4:** Comparison of effects on training on mean communication and teamwork score (secondary outcome)

Element	Total (N = 41)	Mini-course training (N = 31)	Usual training (N = 10)	P-value
**Secondary outcome: Mean communication skills score**				
Pre-intervention	9.94 ± 0.87	9.76 ± 0.72	10.50 ± 1.08	0.02
2-week	12.34 ± 1.35	12.76 ± 1.12	11.05 ± 1.19	< 0.01
16-week	13.43 ± 1.56	13.97 ± 1.24	11.75 ± 1.21	< 0.01
**Subdomain: Giving information clearly and concisely**				
Pre-intervention	2.63 ± 0.39	2.61 ± 0.38	2.70 ± 0.42	0.54
2-week	3.20 ± 0.43	3.27 ± 0.40	2.95 ± 0.44	0.04
16-week	3.41 ± 0.49	3.56 ± 0.40	2.95 ± 0.44	< 0.01
**Subdomain: Including context and intent**				
Pre-intervention	2.59 ± 0.25	2.52 ± 0.20	2.80 ± 0.26	< 0.01
2-week	3.12 ± 0.40	3.23 ± 0.36	2.80 ± 0.35	0.02
16-week	3.39 ± 0.47	3.55 ± 0.42	2.90 ± 0.21	< 0.01
**Subdomain: Receiving information**				
Pre-intervention	2.57 ± 0.24	2.53 ± 0.18	2.70 ± 0.35	0.05
2-week	3.16 ± 0.32	3.24 ± 0.28	2.90 ± 0.32	0.03
16-week	3.34 ± 0.36	3.42 ± 0.34	3.10 ± 0.32	0.01
**Subdomain: Identifying and tackling barriers to communication**				
Pre-intervention	2.15 ± 0.37	2.10 ± 0.35	2.30 ± 0.42	0.14
2-week	2.87 ± 0.45	3.02 ± 0.38	2.40 ± 0.32	< 0.01
16-week	3.28 ± 0.45	3.44 ± 0.36	2.80 ± 0.35	< 0.01
**Secondary outcome: Mean teamwork skills score**				
Pre-intervention	10.55 ± 1.09	10.50 ± 1.02	10.70 ± 1.36	0.62
2-week	12.65 ± 1.40	13.10 ± 1.11	11.25 ± 1.27	< 0.01
16-week	13.78 ± 1.48	14.32 ± 1.09	12.10 ± 1.26	< 0.01
**Subdomain: Supporting others**				
Pre-intervention	2.37 ± 0.46	2.23 ± 0.38	2.80 ± 0.42	< 0.01
2-week	3.28 ± 0.34	3.37 ± 0.29	3.00 ± 0.33	0.02
16-week	3.46 ± 0.44	3.60 ± 0.35	3.05 ± 0.44	< 0.01
**Subdomain: Solving conflicts**				
Pre-intervention	2.60 ± 0.34	2.66 ± 0.30	2.40 ± 0.40	0.03
2-week	3.01 ± 0.43	3.11 ± 0.38	2.70 ± 0.42	0.01
16-week	3.30 ± 0.43	3.44 ± 0.36	2.90 ± 0.39	< 0.01
**Subdomain: Exchanging information**				
Pre-intervention	2.80 ± 0.32	2.81 ± 0.31	2.75 ± 0.35	0.63
2-week	3.21 ± 0.42	3.34 ± 0.35	2.80 ± 0.35	< 0.01
16-week	3.48 ± 0.42	3.60 ± 0.35	3.10 ± 0.39	< 0.01
**Subdomain: Coordinating activities**				
Pre-intervention	2.79 ± 0.33	2.81 ± 0.31	2.75 ± 0.42	0.65
2-week	3.15 ± 0.41	3.27 ± 0.28	2.75 ± 0.49	< 0.01
16-week	3.54 ± 0.47	3.69 ± 0.40	3.05 ± 0.28	< 0.01

NTS: non-technical skills.

The mean teamwork skills score was significantly better in the mini-course training group versus the usual training group both 2 weeks and 16 weeks post-intervention (13.10 ± 1.11 vs. 11.25 ± 1.27; P < 0.01, 14.32 ± 1.09 vs. 12.10 ± 1.26; P < 0.01, respectively). The teamwork skills subdomains (supporting others, solving conflicts, exchanging information, and coordinating activities) showed significant better mean teamwork skill scores after 2 weeks and 16 weeks in the mini-course training group than in the usual training group (Table 4).

**Fig. 2:**
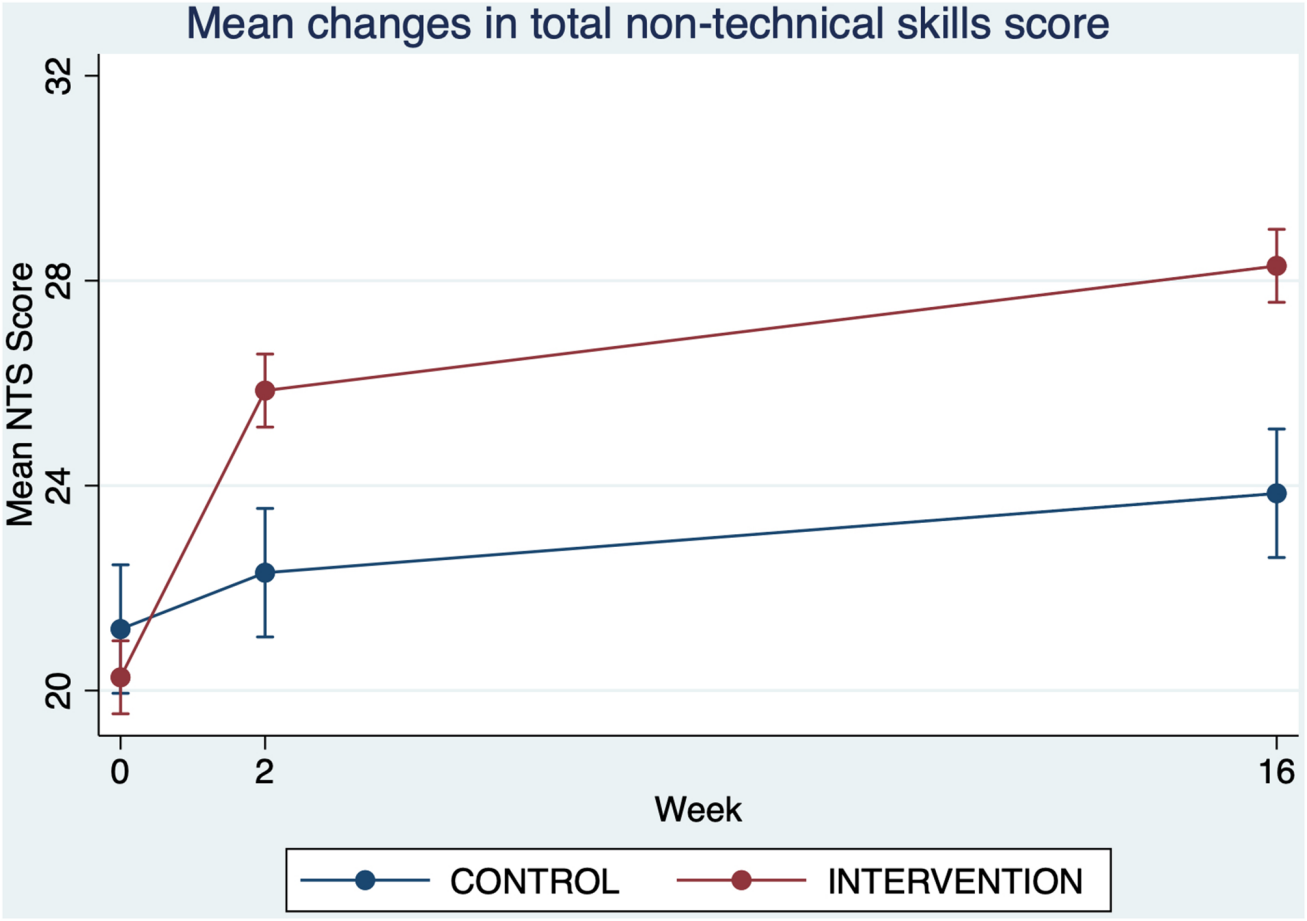
Mean changes in total non-technical skills score (pre-intervention and 2 weeks and 16 weeks post-intervention)

**Fig. 3:**
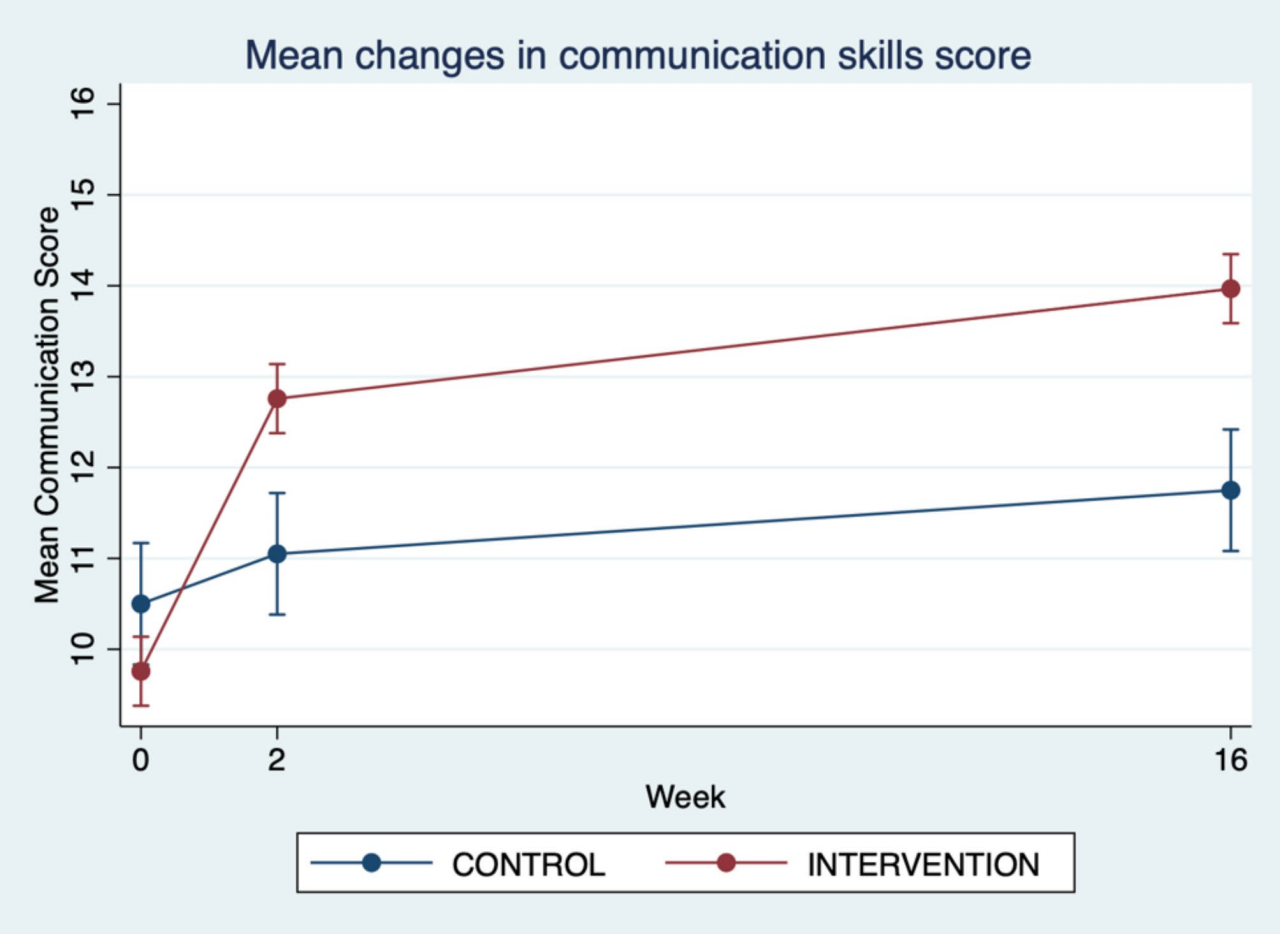
Mean changes in communication skills score (pre-intervention and 2 weeks and 16 weeks post-intervention)

**Fig. 4:**
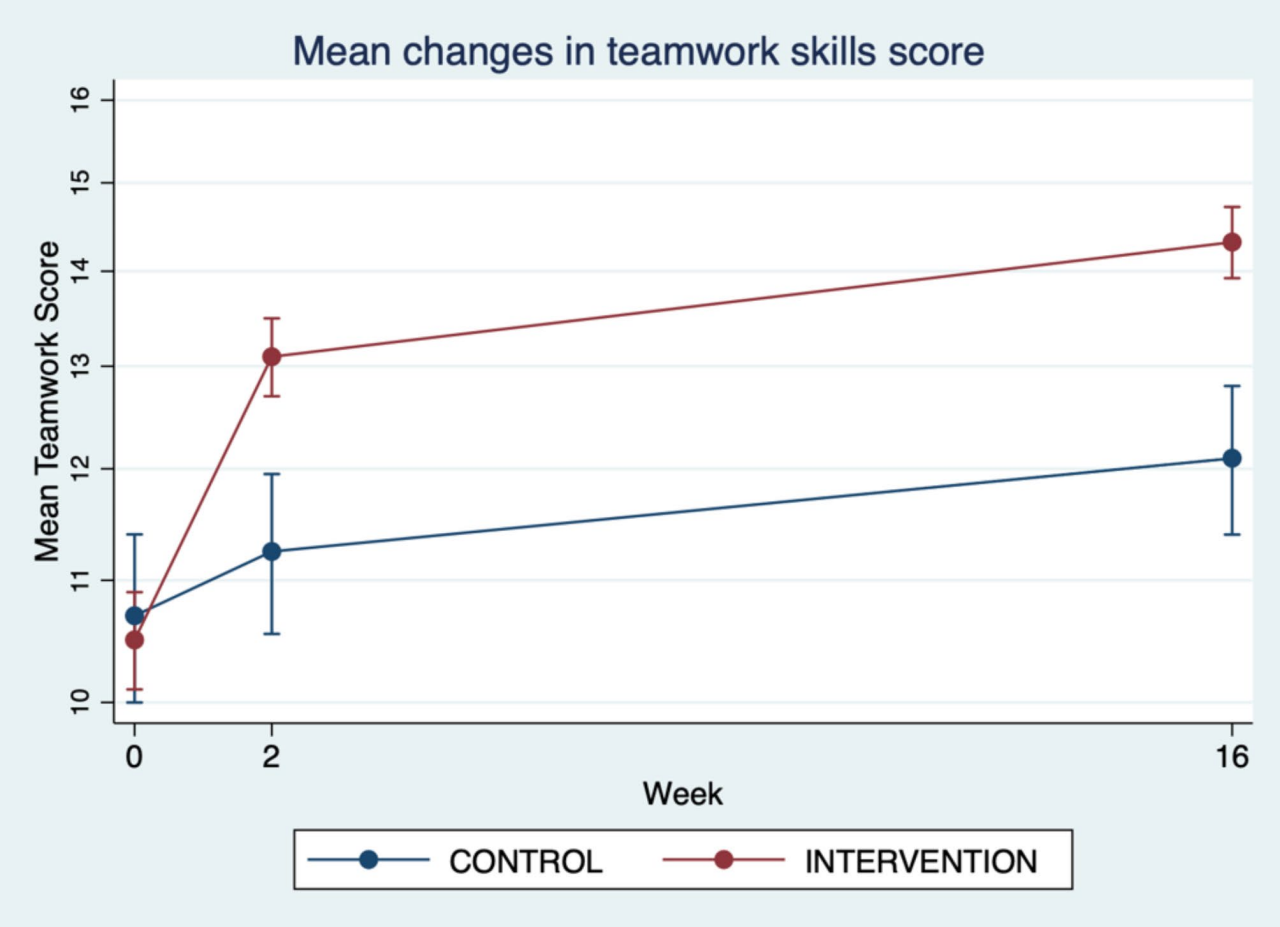
Mean changes in teamwork skills score (pre-intervention and 2 weeks and 16 weeks post-intervention)

Figures 2 and 3, and 4 demonstrate the relationships between the mini-course training group and the usual training group by intervention over time, showing mean changes in total non-technical skills score, mean communication skills score and mean teamwork skills score.

Table 5 demonstrates that the mean total NTS scores at the start of the study were not different by group (coefficient [coef.] 0.94 [95% CI: -0.50–2.38, P = 0.20]). Over time, each group’s NTS score increased 0.15 points every week (95% CI: 0.01–0.28, P = 0.03); however, the mini-course training group showed a greater increase over usual training, 0.24 points after adjustment for time (95% CI: 0.08–0.39, P < 0.01).

**Table 5:** Comparison between mini-course training and usual training with time interaction of mean NTS score

	Coefficient	Confidence interval	P-value
**Mean total NTS score**			
NTS workshop /usual training	0.94	-0.50–2.38	0.20
Time (week)	0.15	0.01–0.28	0.03
Intervention with time	0.24	0.08–0.39	< 0.01
**Mean communication skills score**			
NTS workshop /usual training	0.28	-0.49 − 1.05	0.48
Time (week)	0.07	0.00–0.14	0.07
Intervention with time	0.13	0.05–0.21	< 0.01
**Mean teamwork skills score**			
NTS workshop /usual training	0.66	-0.09–1.40	0.08
Time (week)	0.08	0.01–0.15	0.03
Intervention with time	0.11	0.03–0.19	0.01

NTS: non-technical skills.

At the start of the study, the mean communication score and mean teamwork score were not different between groups (coef. 0.28 [95% CI: -0.49–1.05, P = 0.48] and coef. 0.66 [95% CI: -0.09–1.40, P = 0.08], respectively). Over time, every week, the score of each group increased by 0.07 points (95% CI: 0.00–0.10, P = 0.07) for mean communication score and 0.08 points (95% CI: -0.01–0.15, P = 0.03) for mean teamwork score. The mini-course training group showed a greater increase over time than with usual training, 0.13 points (95% CI: 0.05–0.21, P < 0.01) for mean communication score and 0.11 points (95% CI: 0.03–0.19, P = 0.01) for mean teamwork score.

## Discussion

NTS is a new trend in medical education, including in emergency residents training programs. NTS plays a role in improving outcomes and patient safety [1, 2]. We found it to be a modifiable skill: although NTS scores improved over time without intervention, the mini-course training that we conducted showed significant reinforcement for improving NTS.

At the start of the study, both participant groups shared similar baseline characteristics and NTS scores. The study revealed that at 2 weeks and 16 weeks after the mini-course NTS training, the intervention group’s NTS scores were significantly better than those of the usual training group.

The improved NTS scores 2 weeks after the mini-course NTS training may be because the training demonstrated what good communication and teamwork looked like, effectively coaching residents on how to manage situations and improve themselves. This immediate improvement in skill has been reported after communication workshops in emergency medicine [15, 18] as well as in surgical resident training programs in the operating room [17].

Furthermore, we found emergency residents in the mini-course training group had continuously increased NTS scores in both communication and teamwork skills at 16 weeks after intervention. This score increase appears to directly result from both the intervention and time. The continuous improvement of NTS in this study may well be the result of participants applying NTS to practice, as observed in a study on teaching emotional intelligence in emergency medicine residents, which had a positive effect over time on improving emotional intelligence [16].

As a result, we emphasize that attention should be paid to implementing NTS in the training curricula of emergency residents. Continuous and facilitative feedback by the attending staff may further motivate the residents to improve themselves. The potential challenges or barriers to incorporating NTS training into emergency residents training programs are the need for more time in daily practice, a private environment, dedicated resources, and limited training and mentorship. However, NTS are modifiable, and emphasizing them results in better clinical outcomes. NTS have been shown to affect many clinical outcomes in recent studies; for example, better NTS have been reported to have positive relationships with technical performance during stressful CPR [7, 19] and to increase patient satisfaction [15]. Further studies should be conducted to evaluate clinical outcomes after training in NTS.

### Limitations

This study has some limitations. First, the inter-rater reliability of the two observers showed only Fair agreement (Cohen’s kappa was 0.4, agreement 68.75%). Consequently, we used the mean average between the two observers to represent the NTS score. We suggest that further studies should develop assessment tools to measure NTS that are adapted to be suitable for specific specialties or institutions. For instance, TEAM may be more appropriate for medical emergency teams [4], and AS-NTS for anesthesiology students [8]. Second, our study was conducted in a single university-affiliated tertiary care hospital, which may limit its generalizability. Third, participants were not randomized but classified into the intervention group according to the convenience of the participants, which may have led to selection bias. The sample size of the control group was 10 which below than calculate. However, we calculated the power of statistic from mean NTS score from both groups, the power was 0.819 (more than 80%). Fourth, the study design allowed an excessive amount of time between allocation and intervention, which may have introduced immortal time bias. Fifth, the investigation team and the evaluating staffs were apart of the academic staff who had formal routine feedback for residents 2 times per year (both intervention group and control group) which may introduce information bias. Also, we had limitations in controlling daily feedback from other academic staff by chance. Finally, our study did not present any effects on the quality of patient care or clinical outcomes due to the intervention or increased NTS.

## Conclusions

After adjustment for time interaction, a comparison of NTS score before and 2 weeks and 16 weeks after an NTS mini-course on communication and teamwork skills reveals that this training was associated with improved mean NTS score, mean communication score, and mean teamwork score. We encourage those involved in developing emergency residents curricula and associated healthcare providers to consider implementing NTS training.

## Data Availability

The data supporting this study’s findings are openly available in Harvard Dataverse: “Effect of Mini-course Training Communication and Teamwork on Non-technical skill score in emergency resident: A Prospective experimental study”, 10.7910/DVN/QUCOIR.

## List of abbreviations

AS-NTS: Anesthetists’ Non-Technical Skills
CI: confidence interval
Coef: coefficient
NOTSS: Non-Technical Skills for Surgeons
NTS: Non-technical Skills
TEAM: Team Emergency Assessment Measure
